# Case Report: Painful left bundle branch block syndrome complicated with vasovagal syncope

**DOI:** 10.3389/fcvm.2024.1438320

**Published:** 2025-01-08

**Authors:** Jiangying Luo, Yajun Xue, Fang Liu, Jing Yang, Boda Zhou, Ping Zhang

**Affiliations:** Department of Cardiology, Beijing Tsinghua Changgung Hospital, School of Clinical Medicine, Tsinghua University, Beijing, China

**Keywords:** painful left bundle branch block syndrome, vasovagal syncope, stress echocardiography, head-up tilt test, case report

## Abstract

**Background:**

Painful left bundle branch block (LBBB) syndrome is an uncommon disease that is defined as intermittent episodes of angina associated with simultaneous LBBB changes on an electrocardiogram (ECG) with the absence of flow-limiting coronary artery disease or ischemia on functional testing. Vasovagal syncope (VVS) is the most common cause of syncope and can be provoked by sublingual nitroglycerin (NTG). Herein, we report a case of painful LBBB syndrome complicated with VVS, which was misdiagnosed as acute coronary syndrome and cardiogenic shock.

**Case summary:**

A 62-year-old woman presented with intermittent exertional chest pain for 3 years and deteriorated for 2 weeks. An ECG, transthoracic echocardiography, and laboratory test results were all normal. Exercise treadmill testing induced chest pain, accompanied by new-onset LBBB. She fainted after finishing the test and receiving sublingual NTG, with a rapid decline in heart rate and blood pressure, which was relieved by 0.5 mg of atropine administered intravenously. Coronary angiography showed no evidence of obstructive lesions. Isoprenaline stress echocardiography induced chest pain and rate-dependent LBBB and showed interventricular/intraventricular desynchrony simultaneously. A head-up tilt test verified mixed VVS in the provocative phase. A diagnosis of painful LBBB syndrome complicated with VVS induced by sublingual NTG was made. The patient received an extended-release metoprolol succinate tablet and had no symptoms at a 1-year follow-up.

**Conclusion:**

Painful LBBB syndrome is an uncommon cause of chest pain and is often overlooked by physicians. Misdiagnosis and mistreatment of painful LBBB syndrome may even cause secondary damage, such as VVS induced by sublingual NTG, which is usually used to alleviate angina.

## 1 Introduction

Painful left bundle branch block (LBBB) syndrome is a rarely diagnosed chest pain syndrome caused by intermittent LBBB in the absence of myocardial ischemia (MI), characterized by the abrupt onset of chest pain coinciding with the development of LBBB and simultaneous resolution of symptoms with the resolution of LBBB. The rate dependence of painful LBBB syndrome makes it a frequent confounder of angina as it often occurs during physical exertion. Dyssynchronous ventricular contraction during LBBB seems to be the origin of the pain, which can be evaluated by stress echocardiography. Vasovagal syncope (VVS) is the most common cause of syncope and can be provoked by sublingual nitroglycerin (NTG) during a head-up tilt test (HUTT). Herein, we report a case of painful LBBB syndrome accompanied by VVS induced by sublingual NTG, which was originally used to relieve the patient’s chest pain.

## 2 Case report

### 2.1 Case presentation

**Figure 1:**
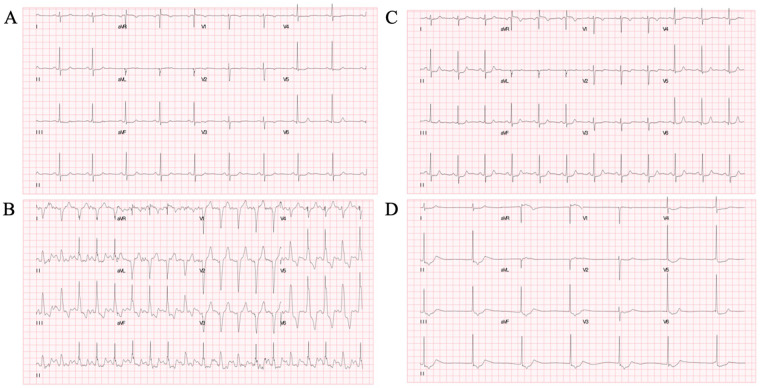
Exercise treadmill test. **(A)** Baseline ECG. **(B)** New-onset LBBB at an HR of 109 bpm in stage 4, correlating with the patient’s typical symptoms of chest pain. **(C)** LBBB disappeared as HR decreased to 71 bpm. **(D)** Syncope after terminating the exercise test at 8 min with a junctional escape rhythm at an HR of 40 bpm and BP 80/50 mmHg after administration of 0.5 mg of sublingual NTG. ECG, electrocardiography; LBBB, left bundle branch block; HR, heart rate; BPM, beats per minute; BP, blood pressure; NTG, nitroglycerin.

A 62-year-old woman presented with intermittent exertional chest pain for 3 years, accompanied by sweating and dizziness, which was relieved within 1–2 min with Wenxin grain. The pain had deteriorated for 2 weeks and was occurring daily. She had a medical history of hypertension and remote cerebral infarction, and she was a non-smoker. Physical examination, a resting electrocardiogram (ECG), transthoracic echocardiography (UCG), and laboratory test results were normal. Exercise treadmill testing (modified Bruce protocol) was performed, and the patient presented with chest pain in stage 4 with ECG monitoring, indicating a new onset of LBBB at a heart rate (HR) of 109 bpm (Figure 1B). With angina pectoris suspected, the test was terminated and the patient received 0.5 mg of sublingual NTG. LBBB disappeared when her HR decreased to 71 bpm (Figure 1C). The patient’s symptoms deteriorated rapidly to dizziness and loss of consciousness, with a junctional escape rhythm at an HR of 40 bpm and blood pressure (BP) declining from 142/80 mmHg to 80/50 mmHg (Figure 1D). After 0.5 mg of atropine was administered intravenously, her vital signs were restored quickly and the patient recovered consciousness without any symptoms. The repeated ECG after resuscitation was similar to the baseline. Acute myocardial infarction (AMI) and cardiogenic shock (CS) were highly suspected and coronary angiography (CAG) was performed but suggested no evidence of obstructive lesions.

**Figure 2:**
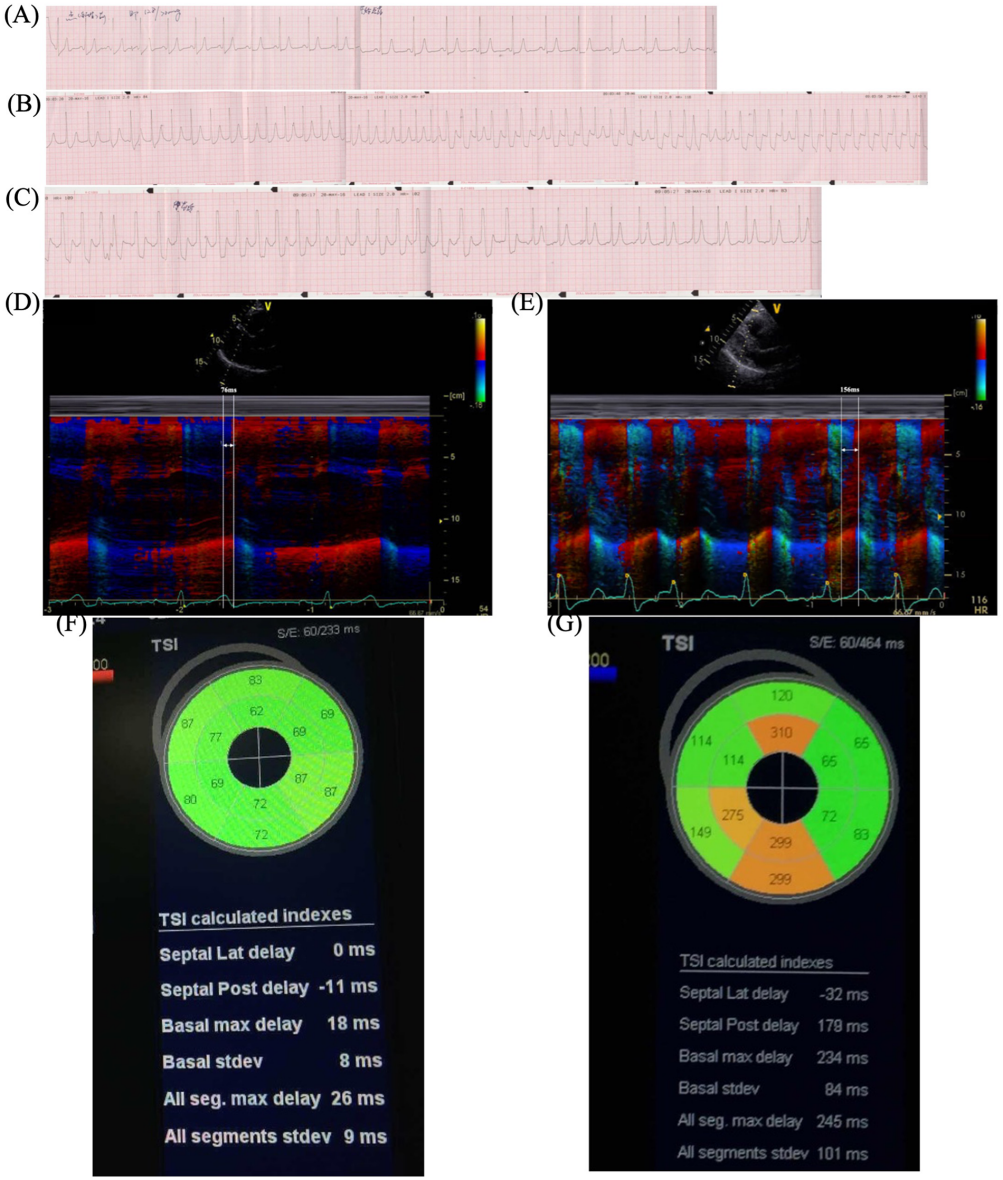
ISO stress UCG test. **(A)** Baseline ECG before ISO. **(B)** New-onset LBBB after ISO when HR increased to 120 bpm and chest pain recurred. **(C)** LBBB and chest pain disappeared when HR decreased to 80 bpm after stopping ISO. **(D)** Color tissue Doppler M-mode UCG showed baseline SPWMD was 76 ms. **(G)** Post-ISO SPWMD increased to 156 ms. **(F)** Baseline TSI. **(G)** Post-ISO TSI. ISO, isoproterenol; UCG, echocardiography; ECG, electrocardiography; LBBB, left bundle branch block syndrome; BPM, beats per minute; HR, heart rate; SPWMD, septal-to-posterior wall motion delay; TSI, tissue synchronization imaging.

To simulate the scenario, isoprenaline (ISO) stress UCG was performed. The continuous ECG monitoring showed the new-onset LBBB when her HR increased from 60 to 120 bpm, and chest pain recurred simultaneously (Figures 2A,B). Furthermore, the stress UCG suggested an increase in septal-to-posterior wall motion delay (SPWMD), anteroseptal-to-posterior wall delay (ΔTS), and aortic pre-ejection interval–pulmonary pre-ejection interval (APEI-PPEI), which indicated interventricular and intraventricular desynchrony (Figures 2D–G, Table 1). The patient’s chest pain and LBBB disappeared when her HR decreased to 80 bpm after terminating ISO (Figure 2C).

**Table 1:** Isoprenaline stress echocardiography test.

	SPWMD (ms)	ΔTS (ms)	APEI-PPEI (ms)
Baseline	76	26	3
Post-ISO	156	245	52

SPWMD, septal-to-posterior wall motion delay; TS, tissue synchronization; APEI-PPEI, aortic pre-ejection interval–pulmonary artery pre-ejection interval.

**Figure 3:**
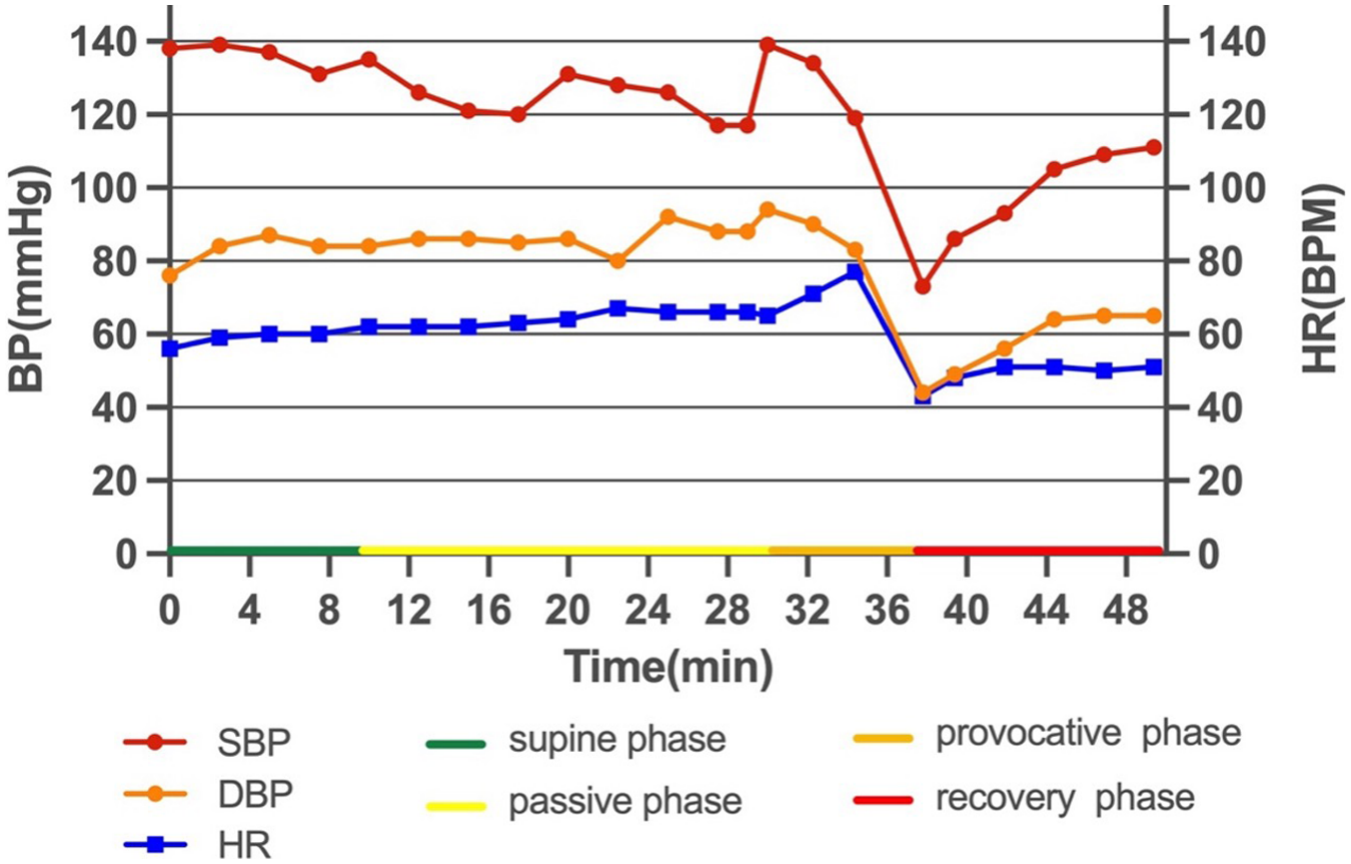
HUTT. The passive phase was negative. Eight min after sublingual NTG in the provocative phase, the patient complained of palpitation and blurred vision, BP decreased from 119/83 to 73/44 mmHg, and HR dropped from 77 to 43 bpm with a sinus rhythm. The patient showed mixed VVS. HUTT, head-up tilt test; NTG, nitroglycerin; BP, blood pressure; HR, heart rate; BPM, beats per minute; VVS, vasovagal syncope.

The patient did not have a quick decline in HR and BP and a loss of consciousness during the stress UCG, which was different from the treadmill test. By reanalyzing the treadmill test result carefully, we found a transient increase in HR (from 71 to 85 bpm) after sublingual NTG and thereafter a quick decline in HR and BP, which corresponded to hemodynamic changes in reflex syncope (Supplementary Material). HUTT was performed and verified mixed VVS in the provocative phase (Figure 3).

Given the evidence, final diagnoses of painful LBBB syndrome and mixed VVS were made. The patient received an extended-release tablet of metoprolol succinate 23.75 mg once a day and presented with no symptoms at a 1-year follow-up.

## 3 Discussion

Given the high mortality rate ascribed to AMI-associated LBBB, new or presumed new LBBB was previously considered diagnostic of AMI. In this case, the patient presented with exertional chest pain, accompanied by new-onset LBBB and suspicious CS so it was reasonable to suspect the diagnosis of AMI. However, this was excluded by the negative CAG, UCG, and cardiac biomarkers. Some studies have shown that more than 50% of patients presenting to the emergency department or chest pain unit with chest discomfort and LBBB are ultimately found to have a diagnosis other than MI (1, 2). Hence, it is important to recognize that the presence of LBBB is not suggestive of ongoing coronary artery occlusion in isolation (2).

Painful LBBB syndrome is a rarely diagnosed chest pain syndrome caused by intermittent LBBB in the absence of myocardial ischemia, characterized by the abrupt onset of chest pain coinciding with the development of LBBB and the simultaneous resolution of symptoms with the resolution of LBBB. In some cases, it can lead to significant physical and psychological debilitation. Vieweg et al. (3) reported the first case of rate-dependent LBBB associated with angina and normal CAG in 1976. Another series of seven similar topics was written in 1982 by Virtanen et al. and two cases were outlined by Said et al. (4, 5) in 2003. Shvilkin et al. (6) developed the following criteria for making the diagnosis of painful LBBB syndrome: (1) abrupt onset of chest pain coinciding with the development of LBBB; (2) the simultaneous resolution of symptoms with the resolution of LBBB; (3) normal 12-lead ECGs before and after LBBB; (4) absence of myocardial ischemia during functional stress testing; (5) normal left ventricular function and the absence of other abnormalities to explain symptoms; (6) low precordial S/T wave ratio consistent with new-onset LBBB and inferior QRS axis.

The mechanisms of painful LBBB syndrome are unclear. Dyssynchronous ventricular contraction during LBBB seems to be the origin of the pain (5). In an intact conduction system, signals rapidly pass through the His bundle to the bundle branches and Purkinje network, which secures synchronized activation of both ventricles. In LBBB, right ventricular activation occurs first, followed by the mid-septum of the left ventricle (LV), with the latest site occurring in the lateral basal area, which causes incoordinate ventricular contractions, decreased contraction efficiency, adverse LV remodeling, and deterioration of mitral regurgitation (7). Studies have verified the compensatory increase in LV filling pressure and therefore higher left atrial (LA) pressure and pulmonary hypertension, which may cause the symptoms of chest pain and dyspnea, as in our patient (7).

Stress UCG can increase HR and evaluate the hemodynamic changes during rate-dependent LBBB. SPWMD of M-mode echocardiography and ΔTS of tissue synchronization imaging (TSI) are useful approaches to quantify intraventricular desynchrony, with the cut-off values of greater than or equal to 130 and 65 ms, separately (8, 9). Interventricular desynchrony can be measured as APEI-PPEI, measured as the time from the onset of the QRS to the onset of pulsed Doppler flow velocities in the LV and right ventricle (RV) outflow tracts respectively, with cut-off values of greater than or equal to 40 ms (9). Consistent with the above, our patient's post-ISO UCG showed significant increases in parameters (SPWMD 76 vs. 156 ms, ΔTS 26 vs. 245 ms, APEI-PPEI 3 vs. 52 ms), which meant interventricular and intraventricular desynchrony during LBBB.

There are no defined treatment protocols for patients with painful LBBB syndrome but some case reports have described the benefits of exercise training, beta-blockers, cardiac resynchronization therapy (CRT), RV pacing, and His bundle pacing (HBP) (6, 10–14). It should be pointed out that CRT and HBP have not been recommended to treat painful LBBB syndrome in the guidelines. CRT is recommended for symptomatic patients with heart failure (HF) in sinus rhythm with left ventricular ejection fraction (LVEF) ≤35%, QRS duration ≥150 ms, and LBBB QRS morphology (Class I; Level A) (15). HBP, alone or optimized in association with coronary sinus pacing (HBP + LV), is proposed as an effective alternative to conventional CRT (16). In CRT candidates for whom coronary sinus lead implantation is unsuccessful, HBP should be considered as a treatment option (Class IIa; Level B) (15). The long-term prognosis of painful LBBB syndrome is generally favorable (14, 17). Some studies reported pain resolution with the development of permanent LBBB (18, 19). No significant adverse events and no heart failure development were observed during the follow-up.

Syncope is defined as transient loss of consciousness due to cerebral hypoperfusion, characterized by a rapid onset, short duration, and spontaneous complete recovery. Syncope is classified as reflex syncope, cardiac syncope, and orthostatic hypotension (20). VVS is the most common cause of syncope. HUTT is a useful diagnostic tool for patients with VVS, owing to its high diagnostic yield with a sensitivity of 66% and a specificity of 89% (21). In this case, the patient first underwent the treadmill test, which activated her sympathetic system, and then sublingual NTG mimicked the pharmacological provocation phase, which induced a transient increase in HR, and then a quick decrease in HR and BP. According to the 2017 ACC/AHA/HRS syncope guidelines (22), beta-blockers might be reasonable in patients 42 years of age or older with recurrent VVS.

The patient received an extended-release tablet of metoprolol succinate and presented with no symptoms at a 1-year follow-up.

## 4 Conclusion

Painful LBBB syndrome is a rare cause of exertional chest pain and should be considered when there is no evidence of coronary artery disease or cardiomyopathies that can explain the symptoms. Misdiagnosis and mistreatment of painful LBBB syndrome may even aggravate the condition and cause secondary damage.

## Data Availability

The raw data supporting the conclusions of this article will be made available by the authors, without undue reservation.
