# Supplementary material for: Case Report: Painful left bundle branch block syndrome complicated with vasovagal syncope
Source: Front Cardiovasc Med. 2025 Jan 8;11:1438320. doi: 10.3389/fcvm.2024.1438320 (PMC11750814; doi:10.3389/fcvm.2024.1438320)
Supplement: Supplementary file 1 [file Image1.pdf]

Supplementary Material

1 Supplementary Figures

(A)

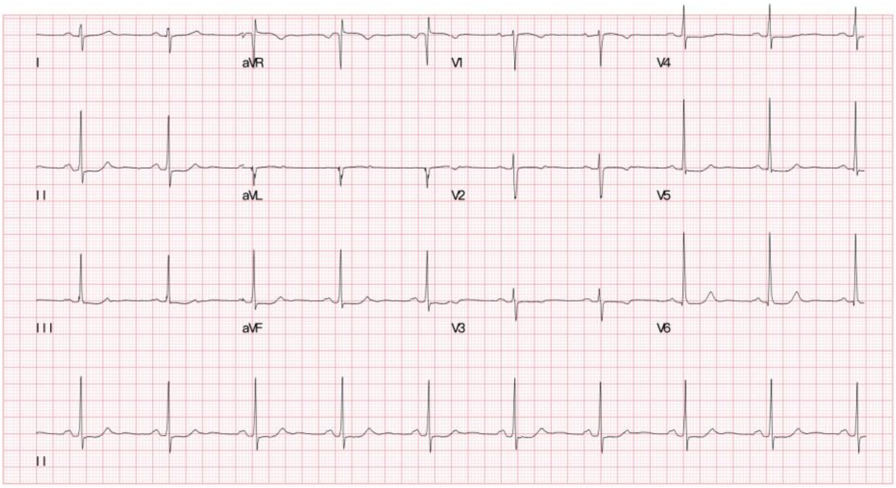

(B)

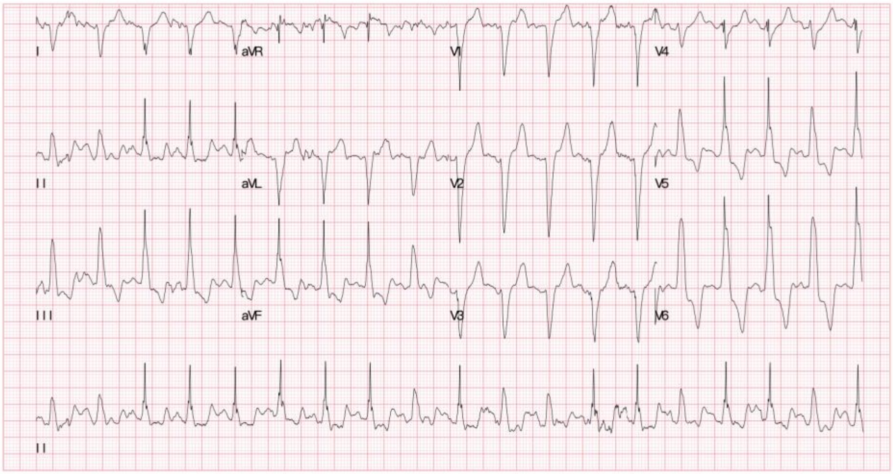

(C)

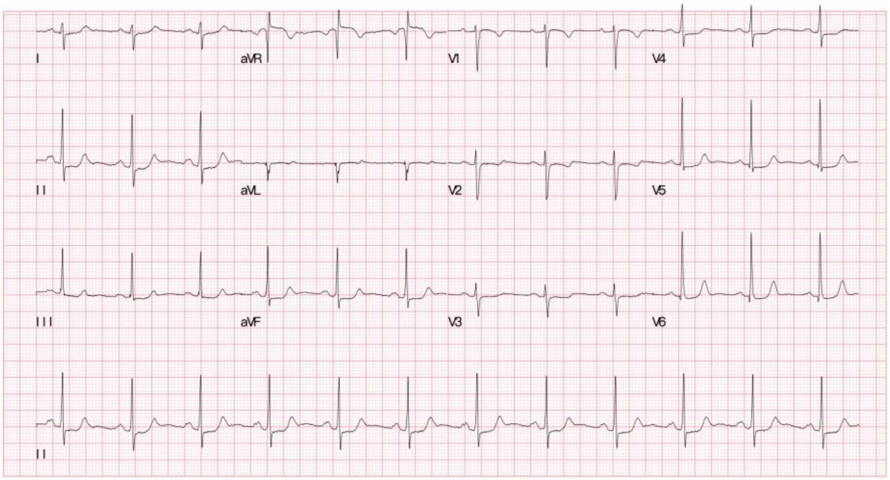

(D)

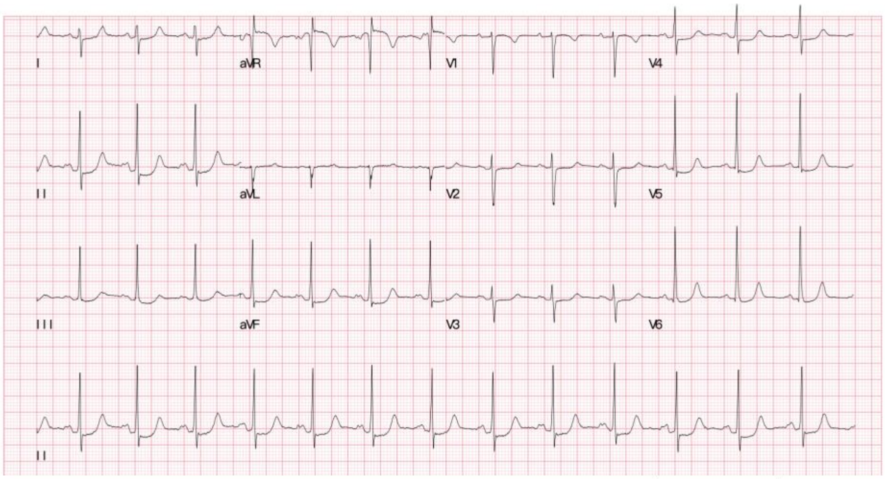

(E)

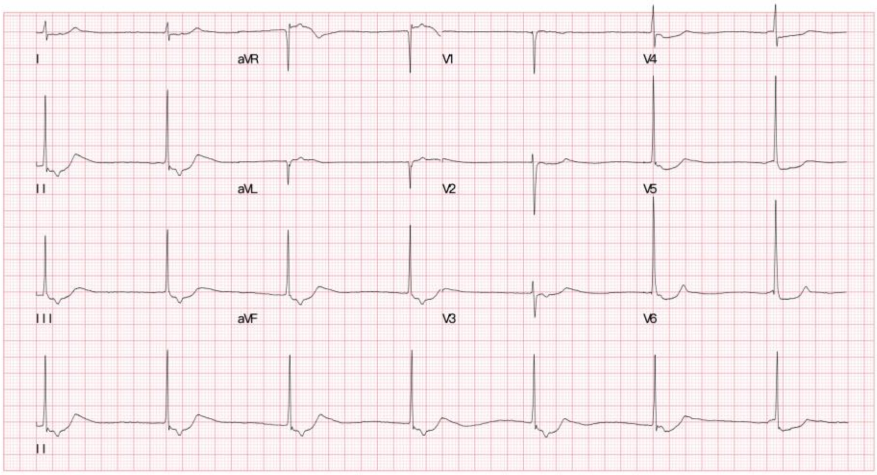

Supplementary Figure 1. Treadmill test exercise. (A) baseline ECG at a HR of 57 bpm. (B) new-onset LBBB at a HR of 109 bpm in stage 4, correlating with chest pain. (C) LBBB disappeared as HR decreased to 71 bpm. (D) HR increased to 85bpm after sublingual NTG 0.5mg. (E) the patient presented with syncope when HR decreased to 40 bpm with junctional escape rhythm and BP decreased from 142/80mmHg to 80/50mmHg.

Abbreviation: ECG, electrocardiography; HR, heart rate; BPM, beats per minute; LBBB, left bundle branch block; NTG, nitroglycerin; BP, blood pressure
